# Liquid Biopsy in Early Screening of Cancers: Emerging Technologies and New Prospects

**DOI:** 10.3390/biomedicines14010158

**Published:** 2026-01-12

**Authors:** Hanyu Zhu, Zhenyu Li, Kunxin Xie, Sajjaad Hassan Kassim, Cheng Cao, Keyu Huang, Zipeng Lu, Chenshan Ma, Ying Li, Kuirong Jiang, Lingdi Yin

**Affiliations:** 1 Pancreas Center, The First Affiliated Hospital of Nanjing Medical University, Nanjing 210000, China; zhuhanyu@stu.njmu.edu.cn (H.Z.); lzy19961216@163.com (Z.L.); xkunxin0617@gmail.com (K.X.); sajjaad15@gmail.com (S.H.K.); drcaocheng@126.com (C.C.); huangkeyu@stu.njmu.edu.cn (K.H.); surgeonmark@hotmail.com (Z.L.); chenshanma2025@gmail.com (C.M.); li_ying03@163.com (Y.L.); 2 Pancreas Institute, Nanjing Medical University, Nanjing 210000, China

**Keywords:** liquid biopsy, cfDNA, CTCs, fragmentomics, multimodal AI, MCED

## Abstract

Liquid biopsy is moving beyond mutation-centric assays to multimodal frameworks that integrate cell-free DNA (cfDNA) signals with additional analytes such as circulating tumor cells (CTCs) and extracellular vesicles (EVs). In this review, we summarize emerging technologies across analytes for early cancer detection, emphasizing sequencing and error-suppression strategies and the growing evidence for multi-cancer early detection (MCED), tissue-of-origin (TOO) inference, diagnostic triage, and longitudinal surveillance. At low tumor fractions, fragmentomic and methylation features preserve tissue and chromatin context; when combined with radiomics using deep learning, they support blood-first, high-specificity risk stratification, increase positive predictive value (PPV), reduce unnecessary procedures, and enhance early prediction of treatment response and relapse. Building on these findings, we propose a pathway-aware workflow: initial blood-based risk scoring, followed by organ-directed imaging, and targeted secondary testing when indicated. We further recommend that model reports include not only discrimination metrics but also calibration, decision-curve analysis, PPV/negative predictive value (NPV) at fixed specificity, and TOO accuracy, alongside multi-site external validation and blinded dataset splits to improve generalizability. Overall, liquid biopsy is transitioning from signal discovery to deployable multimodal decision systems; standardized pre-analytical and analytical workflows, robust error suppression, and prospective real-world evaluations will be pivotal for clinical implementation.

## 1. Introduction

Cancer remains a major global health burden despite advances in prevention, imaging, and therapy; in 2025 there were close to 20 million new cases and almost 9.7 million deaths worldwide, and demographics-based prediction indicates that the number of new cases of cancer will reach 35 million by 2050 [1]. Liquid biopsy offers a minimally invasive window into tumor biology by profiling circulating analytes in blood and other fluids, including cell-free DNA (cfDNA), circulating tumor cells (CTCs), extracellular vesicles (EVs), and RNA/protein markers, spanning applications from multi-cancer early detection (MCED) to surveillance [2]. However, mutation-centric cfDNA assays face structural constraints at the population level. Early disease is characterized by low tumor fractions, and depressing sensitivity at screening-grade specificity, while clonal hematopoiesis introduces background variants that can confound interpretation unless white-blood-cell DNA is co-profiled. These limitations motivate non-mutation signals that better persist at low tumor burden.

The feasibility of cancer detection and targeted intervention relies on the existence of molecular features that reliably distinguish malignant from normal cells. Extensive studies in hematological malignancies have identified cancer-specific or lineage-restricted target antigens, which have enabled the clinical success of CAR T cell therapies and underscore the biological principle that tumors harbor exploitable disease-associated molecular signatures [3]. These signatures are not limited to cell-surface antigens but are also reflected in nucleic acids and vesicular cargo released into the circulation, providing the conceptual foundation for blood-based cancer detection strategies.

Two such signal families have advanced rapidly. Fragmentomics, covering fragment length distributions, end-motif/nuclease preferences, and nucleosome occupancy, captures chromatin and tissue context and enables cancer detection at scale [4]. Epigenomics, especially targeted 5-methylcytosine (5-mC) profiling, has been proved to support MCED with accurate tissue-of-origin (TOO) prediction [5]. Assay design and rigor remain pivotal: pre-analytical handling for cfDNA has been standardized, and error-suppressed sequencing with unique molecular identifiers and duplex consensus improves analytic limits for low-fraction variants [6]. Recent clinical validation further underscores real-world promise: a fragmentome assay demonstrated performance in lung-screening-eligible populations, supporting pre-LDCT triage concepts [7]. A parallel trend is multimodal integration with imaging. Radiomics extracts quantitative descriptors that encode spatial phenotype and organ context, information orthogonal to cfDNA features and well suited for late-fusion via deep learning [8]. Prospective and translational studies now combine CT/radiomics with cfDNA methylation or fragmentomics to improve nodule risk stratification and streamline work-up. For example, an AI model integrating clinical variables, CT features, and cfDNA methylation improved classification of pulmonary nodules [9]. Together, these developments suggest a pathway-aware deployment—from blood-first, high-specificity risk scoring to organ-directed imaging and then targeted secondary testing—to raise positive predictive value (PPV) while reducing unnecessary procedures. This review takes an end-to-end view, synthesizing error-suppression fundamentals, summarizing cfDNA-centric fragmentomic and epigenomic assays alongside complementary analytes, and appraising the evidence for MCED, TOO, diagnostic triage, and surveillance. We also set out reporting and validation practices—including calibration, decision-curve analyses, PPV/NPV at fixed specificity, TOO accuracy, blinded data splits and external validation across sites—that are needed to translate multimodal liquid biopsy into clinical pathways.

## 2. Biological Fluids That Can Be Used in Liquid Biopsy

The human body is largely composed of fluids, which carry diverse molecular information reflecting an individual’s physiological and pathological states. With advances in detection technologies, several biological fluids have been recognized as potential sources for liquid biopsy and explored as clinical tools for early cancer detection, including plasma, urine, cerebrospinal fluid (CSF), and ascites or pleural effusions [10].

In contrast, other body fluids such as serum, whole blood, saliva and feces have also been investigated in earlier studies but are currently limited by biological, technical or analytical constraints that hinder their broad clinical application. In this section, we summarize the major biological fluids used for liquid biopsy and discuss their respective limitations.

### 2.1. Plasma

Plasma refers to the supernatant obtained after anticoagulated whole blood is centrifuged to remove blood cells. Because cellular components—particularly leukocytes—are effectively eliminated, background genomic DNA released from leukocyte lysis is markedly reduced, which facilitates the detection of low-abundance tumor-associated biomarkers such as cfDNA, circulating proteins and extracellular vesicles. Importantly, cfDNA fragmentation patterns and epigenetic features are largely preserved in plasma, maintaining their physiological characteristics. As the circulatory system spans the entire body, molecular signals released from lesions across multiple organs can enter the bloodstream. Consequently, plasma is particularly suitable for integrated analysis of multi-organ tumor signals and has become the most widely used and best-standardized biological fluid in liquid biopsy research [11]. At present, plasma is regarded as the core matrix for multi-cancer early detection and longitudinal disease monitoring.

### 2.2. Urine

Urine is a readily accessible biological fluid that can be collected in a completely non-invasive manner under normal conditions. It generally contains little to no leukocytes, resulting in low levels of genomic DNA background noise, which makes it attractive for certain liquid biopsy applications. However, urine is subject to substantial dilution effects, and cfDNA concentrations can vary considerably depending on hydration status and individual physiological conditions, leading to high inter-sample variability. Moreover, cfDNA fragments in urine are typically short, which limits its utility for fragmentomic and epigenomic analyses [12]. Overall, urine-based liquid biopsy is currently most applicable to tumors of the urinary tract, such as bladder and prostate cancer, whereas its role in population-level pan-cancer early screening remains limited.

### 2.3. CSF

Cerebrospinal fluid constitutes the immediate extracellular environment of the brain and spinal cord and offers unique advantages for liquid biopsy of central nervous system (CNS) tumors. Compared with peripheral plasma, CSF generally contains a higher fraction of tumor-derived cfDNA, enabling sensitive molecular subtyping, detection of resistance mutations and disease monitoring [13]. Nevertheless, CSF acquisition requires invasive procedures such as lumbar puncture or ventricular drainage, which are technically demanding and unsuitable for asymptomatic individuals or routine outpatient screening. In addition, similar to urine, CSF is primarily applicable to specific tumor types rather than broad pan-cancer population screening.

### 2.4. Ascites and Pleural Fluids

In selected clinical contexts, ascites and pleural effusions can serve as highly informative liquid biopsy sources. Because these fluids often originate from or are in close proximity to tumor sites, the abundance of cfDNA and CTCs within them is typically higher than in peripheral blood, providing high sensitivity for molecular detection of thoracic, abdominal and pelvic malignancies, including lung, ovarian and gastric cancers. However, ascites and pleural effusions are usually observed in advanced-stage disease and are rarely available in early cancer. Moreover, their presence is not universal among patients, limiting their applicability for large-scale screening. Non-malignant conditions, such as ascites caused by liver cirrhosis, may also confound result interpretation. Therefore, ascites and pleural fluids are not suitable for early cancer screening and are mainly restricted to specific disease stages and clinical questions.

### 2.5. Serum and Whole Blood

Serum and whole blood present substantial limitations for liquid biopsy compared with plasma. Serum is obtained after natural coagulation of whole blood, during which leukocyte activation and lysis can occur, releasing large amounts of genomic DNA that significantly dilute low frequency cfDNA signals and distort fragmentation patterns [14]. Similarly, whole blood is characterized by high background noise due to leukocyte-derived DNA contamination and is therefore unsuitable for cfDNA-based, fragmentomic or epigenetic liquid biopsy analyses. Consequently, neither serum nor whole blood is generally recommended for cfDNA-focused liquid biopsy applications. It should be noted, however, that whole blood remains the standard sample type for CTC detection. In recent years, rapid advances in CTC enrichment and single-cell analysis technologies have enabled the extraction of rich biological information from whole blood, highlighting its potential value in tumor biology studies and precision oncology.

### 2.6. Saliva

Saliva represents a non-invasive biological fluid with potential applicability in certain cancer types, particularly head and neck squamous cell carcinoma. However, salivary samples are heavily influenced by oral microbiota, food residues and local inflammatory conditions, resulting in high background noise and limited specificity. In addition, standardized pre-analytical and analytical workflows for saliva-based liquid biopsy are currently lacking, which hampers its broader clinical application in systemic cancer detection [15].

### 2.7. Feces

Fecal samples are generally not considered part of the liquid biopsy framework. Although their potential utility for colorectal cancer detection has been explored, digestive processes lead to extensive degradation of tumor-associated biomarkers, and microbial DNA overwhelmingly dominates fecal nucleic acid content [16]. Furthermore, fecal sample composition is highly variable and strongly influenced by individual gastrointestinal conditions, making standardized sampling and processing difficult. As a result, feces are more appropriately classified within specific molecular screening strategies rather than as a viable source for liquid biopsy.

## 3. Analytes and Signals for Liquid Biopsy

**Figure 1:**
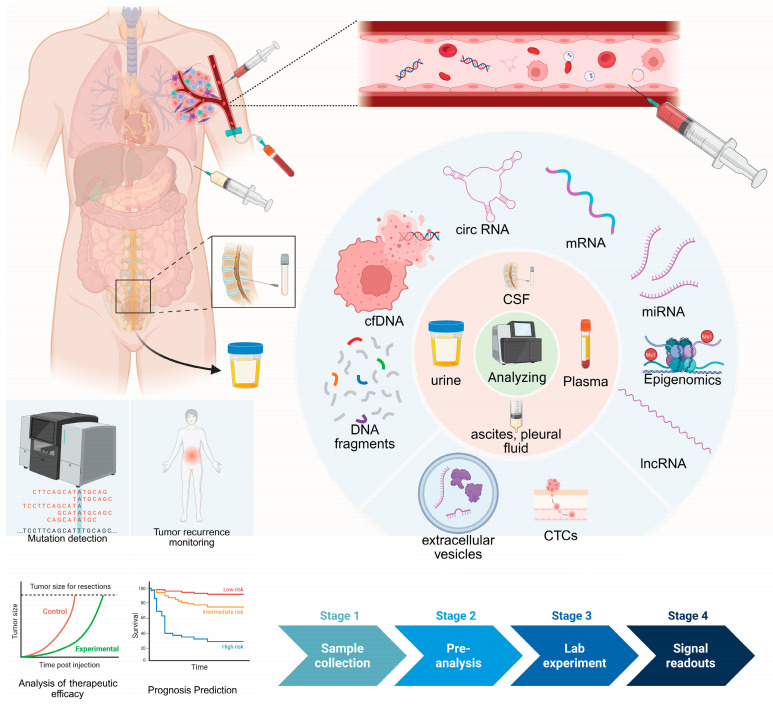
In liquid biopsy, biological fluids such as CSF, urine, blood, and ascites or pleural effusions are collected from patients and subsequently analyzed for tumor-derived biomarkers, including circulating cfDNA, ncRNAs, EVs, and CTCs. Information obtained from fragmentomic and epigenomic analyses can be applied to mutation detection, tumor recurrence monitoring, assessment of therapeutic efficacy, and prognostic prediction.

Multiple liquid-biopsy analytes—including cfDNA, CTCs, EVs, tumor-educated platelets (TEPs), and circulating RNAs, particularly noncoding RNAs (miRNAs, lncRNAs, circRNAs)—show strong associations with cancer (Figure 1).

### 3.1. cfDNA/ctDNA

**Figure 2:**
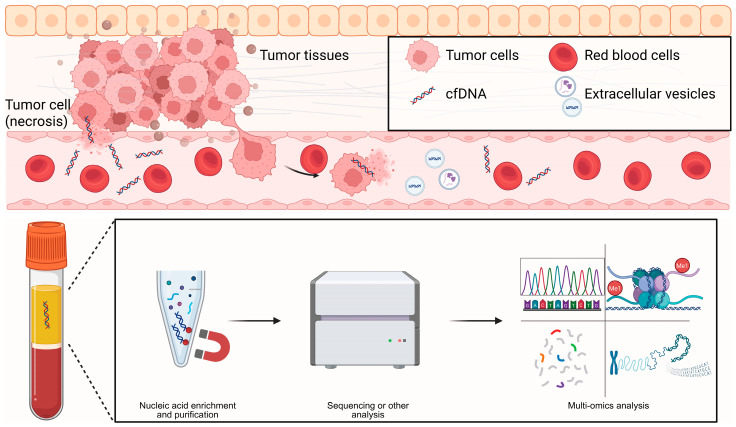
cfDNA consists of short DNA fragments released into the circulation or other biological fluids from primary tumor tissues or from apoptotic and necrotic circulating tumor cells. After isolation from plasma—commonly using methods such as magnetic bead-based purification—cfDNA is subjected to downstream analyses, including sequencing and other molecular assays. Multi-omics approaches, such as fragmentomic and epigenomic analyses, enable the decoding of tumor-associated information embedded within cfDNA.

cfDNA consists of short DNA fragments released into biofluids by dying or stressed cells, through apoptosis, necrosis, NETosis, or active secretion. In 1997, Lo et al. demonstrated that fetal cfDNA in maternal plasma enables non-invasive prenatal testing, establishing a paradigm for cfDNA-based diagnostics [17]. The fragmentation landscape is not random: fragment sizes peak around mono- and oligo-nucleosomal lengths and retain nucleosome footprints that encode tissue of origin. Tumor-derived fragments are typically shorter (often <150 bp) and show characteristic end coordinates and end motifs, all of which provide diagnostic signal (Figure 2) [18]. Kinetically, cfDNA is cleared rapidly from the circulation. Fetal cfDNA has a half-life on the order of minutes, whereas ctDNA more often exhibits hour-scale kinetics, allowing near-real-time pharmacodynamic readouts during therapy [19]. Clinically, cfDNA analysis underpins minimal residual disease (MRD) assessment, treatment monitoring, transplant rejection surveillance, and multi-cancer early detection; we focus here on the latter. Assays span from targeted panels to genome-wide approaches. Beyond somatic mutations, methylation patterns and fragmentomic features improve sensitivity and TOO inference at low tumor fractions. A key caveat is clonal hematopoiesis (CHIP/CH), which generates background variants and must be mitigated using matched white blood cell sequencing or robust computational filtering. Integrating mutations, copy-number alterations, methylation, and fragmentomic features in “multi-omic cfDNA” frameworks have the potential to further strengthen clinical decision-making when tumor fractions are very low [20].

A fundamental limitation of cfDNA-based liquid biopsy is its limited biological specificity. Alterations in cfDNA abundance and fragmentation patterns are not exclusively driven by malignancy but can also arise from age-associated clonal hematopoiesis, chronic inflammation, tissue injury and other non-neoplastic conditions. Increasing age is associated with expanded hematopoietic clones that contribute somatic mutations to circulating DNA, while inflammatory processes can increase cfDNA release through enhanced cell turnover and apoptosis. These non-tumor-derived signals represent a major source of biological confounding, complicating the interpretation of cfDNA abnormalities in cancer screening and early detection settings [11].

Consequently, cfDNA-based screening approaches increasingly rely on orthogonal features, such as fragmentomic profiles, epigenetic patterns and multimodal integration, to mitigate biological confounding and improve tumor specificity.

### 3.2. CTCs

Circulating tumor cells (CTCs) are intact malignant cells shed into the blood or lymphatic circulation, providing whole-cell readouts that are not accessible from acellular analytes [21]. The FDA-cleared CellSearch system standardizes CTC enumeration (events per 7.5 mL), where counts ≥5/7.5 mL are associated with adverse outcomes. In addition, the presence of CTC clusters correlates with higher metastatic potential.

High-content analysis now combines single-cell phenotyping and multi-omics to delineate epithelial–mesenchymal transition (EMT) and immune-evasion programs, with an expanding toolkit of capture technologies. Affinity-based and label-free platforms—including microfluidic devices, negative-selection strategies, and size-based enrichment—extend recovery beyond EpCAM-high epithelial CTCs [22]. Downstream analyses include immunophenotyping; single-cell DNA sequencing to resolve subclonal architecture (SNVs, CNAs, and phylogeny); and single-cell transcriptomic/epigenomic profiling together with multiplex protein/phospho assays to map AR, KRAS, EMT, and interferon-response states and to monitor signaling in near real time. Functional platforms such as ex vivo CTC-derived organoids, xenografts, and invasion or drug-response assays then link these molecular profiles to therapeutic sensitivity and metastatic potential [23].

However, despite substantial advances in CTC isolation and characterization technologies, their clinical applicability in early cancer detection remains fundamentally limited by extremely low detection rates in stage I–II disease. In early-stage cancers, CTCs are often absent or present at frequencies below the detection threshold of current platforms, resulting in poor sensitivity and high false-negative rates. Consequently, most clinical evidence supporting CTC analysis has been derived from patients with advanced or metastatic disease, where tumor burden and vascular shedding are substantially higher [24]. These biological constraints, together with limited scalability and technical complexity, currently preclude CTCs from serving as reliable biomarkers for population-level screening or early-stage cancer detection. Importantly, this limitation reflects a biological constraint rather than a purely technical shortcoming, as early-stage tumors may not consistently release intact cells into the circulation.

### 3.3. EVs

Extracellular vesicles (EVs) are lipid-bilayer nanoparticles released by most cell types. They include exosomes, microvesicles/ectosomes, and apoptotic bodies. Because vesicle biogenesis cannot be directly demonstrated in clinical isolates, the International Society for Extracellular Vesicles (ISEV) recommends using operational definitions based on size and marker profiles, together with transparent reporting standards.

EVs carry diverse cargos, including proteins, lipids, glycans, and nucleic acids. Exosomal RNA transfer (mRNA and miRNA) can be functionally active, and tumor-derived EVs transport oncogenic cargos with diagnostic potential; for example, selected EV-associated microRNAs have been proposed as candidate markers in prostate cancer.

Early reports that double-stranded exosomal DNA (exoDNA) harbors tumor-derived mutations prompted re-evaluation of EV-associated DNA. High-resolution fractionation and direct capture studies now suggest that a substantial fraction of extracellular DNA is non-vesicular, highlighting the need for caution when interpreting EV-based liquid biopsy signals [25].

Each EV isolation method balances yield against purity. Differential ultracentrifugation is widely used but co-isolates lipoproteins and protein complexes. Size-exclusion chromatography typically improves purity and better preserves EV integrity compared with PEG precipitation. Microfluidic platforms, which use immunoaffinity capture, nanostructured surfaces, or acoustofluidics, allow small input volumes, rapid processing, and on-chip detection. Rigorous purity control, including quantification of lipoprotein carryover (ApoB/A1) and explicit depletion of non-vesicular contaminants, can substantially improve biomarker discovery [26,27]. Across multiple tumor types (e.g., NSCLC, melanoma, pancreatic cancer), EV-based biomarkers show encouraging signals; however, several high-profile candidates still require independent replication under careful pre-analytical control.

Currently, a major limitation is that the clinical deployment of extracellular vesicle assays remains challenging. A major limitation lies in the difficulty of isolating pure and well-defined EV populations, as current isolation methods—including ultracentrifugation, size-exclusion chromatography and immunoaffinity capture—often yield heterogeneous vesicle fractions with variable purity. This methodological heterogeneity substantially compromises assay reproducibility and limits cross-study comparability. Moreover, although EVs carry diverse molecular cargo, there is currently limited evidence that EV-based assays provide superior sensitivity or specificity for early cancer detection compared with established cfDNA-based approaches. Most reported EV studies remain exploratory, rely on small cohorts, and lack head-to-head comparisons with cfDNA in prospective screening settings. Consequently, EVs are best regarded at present as complementary analytes rather than replacements for cfDNA in early detection and multi-cancer screening workflows.

### 3.4. TEPs

Tumor-educated platelets (TEPs) are circulating platelets whose RNA—and, to a lesser extent, protein—profiles are remodeled by cancer through direct platelet–tumor contact, uptake of tumor-derived factors and vesicles, and education during megakaryopoiesis. Despite being anucleate, platelets retain pre-mRNAs and a functional spliceosome, enabling stimulus-dependent splicing that dynamically reshapes their transcriptome [28].

Patient-derived platelets from glioma and prostate cancer harbor tumor-derived RNAs, and both in vitro and in vivo studies support transfer of mutant tumor RNA into platelets. RNA sequencing of TEPs can classify tumors by mutational status and infer pathway activity, consistent with ongoing RNA exchange and education rather than passive contamination. Stimulus-induced splicing generates translatable mRNAs and protein products, exemplified by tissue factor and IL-1β.

Tumor- and inflammation-driven cytokines, particularly IL-6, increase thrombopoietin (TPO) production, skew megakaryopoiesis, and enhance platelet reactivity, providing an upstream mechanism by which tumors shape the platelet RNA and proteomic landscape. Together, these processes allow TEPs to enrich tumor-relevant transcripts and provide robust signal even at low tumor burden [29,30]. TEP-derived RNA panels therefore show promise as complements to blood-based cancer screening, encoding both pathway activity and tissue-of-origin information to aid triage. Most current studies on TEPs rely primarily on RNA sequencing, and the methodological scope remains relatively narrow. Therefore, this topic will not be further elaborated in the following sections.

### 3.5. Non-Coding RNA

Noncoding RNAs (ncRNAs) are increasingly supported as informative liquid-biopsy biomarkers by converging mechanistic and clinical evidence. Cells can package functional mRNAs and miRNAs into exosomes, and glioblastoma-derived extracellular vesicles (EVs) have been shown to transport oncogenic RNAs and proteins. In the circulation, miRNAs are resistant to RNase-mediated degradation, can be reliably quantified in serum and plasma, and can distinguish cancer patients from non-cancer controls; this stability is largely attributed to association with Ago2-containing complexes and, in part, to packaging within EVs. Beyond miRNAs, the urinary lncRNA PCA3 has undergone regulatory-grade clinical validation for prostate-cancer risk stratification [31]. Sensitive ncRNA profiling is now routine with RT–qPCR, droplet digital PCR, and small-RNA sequencing. Large clinical studies illustrate their diagnostic value. For example, the 12-miRNA plasma assay GASTROClear achieved area-under-the-curve values around 0.9 for gastric cancer detection in a three-phase multicenter trial (*n* > 5000), and other serum miRNA signatures and UCA1-based assays support non-invasive detection of colorectal and additional cancers [32]. High-throughput RNA sequencing has also established circular RNAs (circRNAs) as abundant, evolutionarily conserved, and RNase R-resistant, with back-splice junction reads providing a robust basis for quantification and quality control [33]. Exploratory studies have reported dysregulation of specific circular RNAs in cancer tissues and matched biofluids, suggesting their potential as auxiliary biomarkers. For example, circ_0004592 was reported to be upregulated in gastric cancer (GC) tissues and plasma, with plasma levels increasing from healthy controls to patients with precancerous lesions and reaching the highest levels in GC. In the same single-center study, postoperative plasma circ_0004592 decreased in paired samples and higher circ_0004592 levels were associated with tumor differentiation, invasion depth, and lymph node metastasis. However, these observations were generated from a case–control design (GC *n* = 100; healthy controls *n* = 100; benign lesions *n* = 15; precancerous lesions *n* = 15) and should be interpreted as exploratory. In that cohort, plasma circ_0004592 achieved an AUC of 0.787 (95PD CI 0.719–0.854) for distinguishing GC from healthy donors, with 66.0% sensitivity and 84.0% specificity at the reported cut-off; combining circ_0004592 with conventional markers (CEA and CA19-9) increased the AUC to 0.831 (95% CI 0.773–0.889) and sensitivity to 85.0%, but reduced specificity to 65.0%. Importantly, unlike cfDNA-based approaches, circRNA biomarkers such as circ_0004592 generally lack large-scale, prospective validation and screening-grade clinical evidence, and robust head-to-head comparisons against established cfDNA assays remain limited. In pancreatic ductal adenocarcinoma (PDAC), a separate circRNA risk-score panel demonstrated improved diagnostic performance when combined with CA19-9 (reported AUC 0.94 in a validation cohort), illustrating the potential complementarity of circRNA signals with existing serum markers; nevertheless, these data are likewise derived primarily from case–control cohorts and require broader prospective evaluation before routine clinical implementation [34,35].

For population-level early detection, however, cfDNA currently dominates because genome-wide methylation and fragmentomic signals retain sensitivity at ultra-low tumor fractions and support TOO inference. CTCs, EVs and other analytes lack cross-site standardization and prospective return-of-results evidence at fixed high specificity, so they are best positioned today as complements to cfDNA within multimodal pipelines (Table 1) [36]. With technological advances and rigorous prospective validation, some may gain larger roles [37].

## 4. Assay Platforms for cfDNA

Here, we summarize representative detection and operational workflows currently used for cfDNA (Table 2).

### 4.1. ULP/Low Pass WGS for CNAs and Fragmentomics

Whole-genome sequencing (WGS) of cfDNA can be performed without prior capture or targeting. By sequencing depth, WGS is often divided into shallow WGS (sWGS) and deep WGS. Deep WGS can profile genome-wide SNVs, indels, structural variants (SVs), and copy-number alterations (CNAs), but generating reliable calls at variant allele frequencies (VAFs) below ~0.5% requires UMI- or duplex-based error suppression, pushing sequencing depth, cost, and data burden into a research-oriented range [38]. In contrast, sWGS samples the genome at low depth yet performs well for genome-wide CNA profiling, tumor fraction (TFx) estimation, and fragmentomic analysis. These fragmentomic signatures have recently emerged as useful biomarkers for cancer detection and TOO inference. Thus, sWGS is best viewed as a complementary approach that provides genome-wide surveillance rather than precise mutation calling, whereas deep WGS supports complex SV discovery or exploratory designs [10].

### 4.2. WGBS/Enzymatic 5-mC Assays

Whole-genome bisulfite sequencing (WGBS) generates base-resolution methylomes by converting unmethylated cytosines to uracil while preserving 5-mC, enabling quantitative analysis across millions of CpG sites. Aberrant CpG-island methylation is a recurrent hallmark of cancer, and cfDNA-based WGBS can support detection, TOO inference, and longitudinal monitoring even at low TFx. The trade-off is that bisulfite chemistry can induce DNA degradation, and incomplete conversion may introduce systematic errors and information loss [39].

### 4.3. Digital PCR (ddPCR/BEAMing) for Hotspot Variants

Digital droplet PCR (ddPCR) partitions each reaction into ~10^4^–10^5^ droplets, with probe-based binary readout and Poisson statistics for absolute quantification, enabling limits of detection around 0.01–0.1% variant allele frequency in optimized assays. BEAMing (beads, emulsion, amplification, magnetics) performs PCR on bead templates within an emulsion; each bead carries clonal amplicons that are subsequently hybridized with mutation-specific probes and counted by flow cytometry, achieving similar analytical sensitivity and, for selected hotspots, limits of detection as low as ~0.02–0.04% [40,41]. Both methods require a priori specification of targets, which constrains multiplexing and genomic breadth, and PCR errors arising in early cycles can be clonally propagated within individual droplets or beads. Their workflows, particularly for BEAMing, are more complex and instrumentation-intensive than panel-based NGS. Clinically, ddPCR and BEAMing provide highly specific, quantitatively robust measurements with good concordance in targeted applications, but they are not suitable for broad genomic profiling.

### 4.4. Amplicon NGS (Tam-Seq and Derivatives)

Tagged-amplicon deep sequencing (TAm-Seq) ligates adapters and unique molecular identifiers (UMIs) to native cfDNA fragments, enabling deep amplicon sequencing with built-in error suppression and high sensitivity at low DNA abundance. Limitations include primer-site dropout, uneven coverage in complex or GC-rich loci, and inherently constrained genomic breadth relative to capture-based methods, which reduces its suitability for comprehensive profiling [11].

### 4.5. CAPP-Seq

Cancer Personalized Profiling by deep Sequencing (CAPP-seq) uses hybrid capture combined with UMI-aware background suppression to detect SNVs/indels, copy-number alterations, and gene fusions from a single cfDNA library and to estimate tumor fraction. Analytical sensitivity ultimately scales with the number of unique cfDNA molecules sampled, which is determined by input DNA amount and library complexity. Capture-related biases, particularly at GC-rich loci, and confounding by CH can generate false or ambiguous variants, necessitating matched white blood cell (WBC) sequencing or stringent bioinformatic filters [42].

### 4.6. WES

cfDNA-based whole-exome sequencing (WES) surveys coding regions where most pathogenic variants reside, enabling variant discovery, tracking of clonal evolution, and therapy guidance, with reports of resistance mutations being detected before radiographic progression. In population or early-detection screening settings, however, very low tumor fraction substantially limits sensitivity, and the sequencing depths required for adequate performance drive up cost and analytic complexity [43]. CH further confounds attribution of variant origin in the absence of matched WBC sequencing. Error-suppressed deep WES and integration with additional omics layers can improve accuracy, but these approaches remain resource-intensive.

## 5. cfDNA for Early Detection: Current Clinical Use and the MCED Pathway

In tumor-naïve population screening, assays that aggregate dense, genome-wide features are favored, because methylation and fragmentomic profiles can transform sparse mutation signals into millions of independent measurements. Whole-genome or targeted methylome profiling captures cancer-associated hyper- and hypomethylation, as well as shifts in CpG context, that persist even at ultra-low tumor fractions and encode tissue of origin, as demonstrated in the Circulating Cell-free Genome Atlas (CCGA) study [44,45]. Fragmentomic analyses exploit differences in fragment size distributions, end motifs, and nucleosome phasing that reflect underlying chromatin architecture, thereby boosting sensitivity and providing orthogonal information for TOO assignment. Low-coverage copy-number/aneuploidy profiling adds a tumor-wide signal that is robust at low sequencing depth, albeit with more limited TOO resolution [46]. Comparative evaluations consistently indicate that methylation- and fragmentomics-based approaches achieve lower clinical limits of detection for multi-cancer early detection (MCED) than mutation-only assays, while integrated models that combine these features further optimize sensitivity and TOO accuracy. Here we summarize some representative clinical and translational studies supporting these approaches (Table 3).

### 5.1. Fragmentomics from Shallow WGS

#### 5.1.1. DELFI and Genome-Wide Fragmentation Profiles

DELFI (DNA Evaluation of Fragments for Early Interception) quantifies position-dependent imbalances in fragment size and coverage using low-depth WGS (~0.5–2×). In the foundational Nature study, it achieved an AUC of 0.94 with ≥95% specificity and 79% sensitivity in resectable (stage I–III) cancers, with preliminary tissue-of-origin (TOO) signals [47]. In lung cancer, the prospective LUCAS study showed strong discrimination (AUC 0.90–0.94) and stage-specific separation; histology-aware features even distinguished SCLC from NSCLC via ASCL1-proximal fragmentation (AUC 0.98). A modeled DELFI + LDCT pathway increased specificity to ~80% while maintaining high sensitivity (overall 90–94%; stage I 80–87%) [48].

**Table 3:** Representative clinical and translational studies of liquid biopsy for early cancer detection.

Study/Trial Name	Type	Cancer Types and Stage Coverage	Analytes	Methods	Reported Performance and Key Findings
cfDNA Methylation MCED Validation (CCGA) [20]	Large-scale clinical validation study (case–control design; development and validation cohorts)	>50 cancer types; stages I–IV (including early-stage cancers)	Plasma cfDNA methylation patterns	Targeted cfDNA methylation sequencing combined with machine-learning–based classifiers for cancer signal detection and TOO prediction	High specificity (~99.5%); overall sensitivity varied substantially by cancer type and stage, with lower sensitivity in stage I–II disease and increasing sensitivity at later stages; high accuracy for cancer signal origin among detected cases
PATHFINDER [49]	Prospective, interventional, multicenter cohort study (clinical implementation study)	Multiple cancer types; screening population without known cancer at enrollment	Plasma cfDNA methylation signatures	Blood-based MCED test using cfDNA methylation profiling; results returned to clinicians with downstream diagnostic evaluation embedded within a clinical diagnostic pathway	High specificity (>99%); cancer detection rate consistent with prior validation studies; demonstrated feasibility of integrating MCED testing into real-world clinical workflows and diagnostic pathways
cfDNA Fragmentation Biology Study [4]	Observational translational research study (methodological and biological characterization)	Multiple cancer types; predominantly patients with established cancer versus healthy controls	Plasma cfDNA fragmentation patterns (fragment size, genomic distribution)	Low-coverage whole-genome sequencing of cfDNA to analyze fragmentation profiles and nucleosomal patterns; primarily focused on biological and technical characterization rather than clinical application	Demonstrated reproducible differences in cfDNA fragmentation patterns between cancer and non-cancer samples; provided biological and technical rationale for fragmentomics-based detection, without reporting screening-grade sensitivity or specificity
Lung Cancer Fragmentome Validation [7]	Clinical validation study with independent cohorts	Lung cancer; screening-eligible and clinically relevant populations, including early-stage disease	Plasma cfDNA genome-wide fragmentation features (fragmentome)	Low-coverage whole-genome cfDNA sequencing with machine learning–based classification of fragmentation features; designed to augment lung cancer early detection strategies	Fragmentation profiles reflected lung cancer–associated chromatin and genomic features; demonstrated high sensitivity and consistent performance across demographic subgroups and common comorbidities; positioned as an adjunct to lung cancer screening strategies
Blood Test and PET-CT Screening Study [50]	Prospective population-based interventional study	Multiple cancer types; asymptomatic individuals undergoing routine health screening	Plasma cfDNA (targeted mutation analysis) combined with imaging findings	Blood-based molecular cancer screening followed by PET–CT imaging for lesion localization and confirmation	Blood testing identified individuals at increased cancer risk; combined molecular screening and imaging enabled detection of multiple cancers with acceptable false-positive rates in a real-world screening setting
SYMPLIFY [51]	Prospective, multicenter observational study	Multiple cancer types; patients presenting with non-specific but concerning symptoms	Plasma cfDNA methylation patterns	Blood-based MCED test with methylation profiling and cancer signal origin prediction; evaluated alongside standard diagnostic pathways	Overall sensitivity approximately two-thirds across cancer types; sensitivity increased with advancing stage; high accuracy for cancer signal origin prediction in detected cases

#### 5.1.2. Orientation-Aware Ends and Nucleosome Footprints

cfDNA fragment ends cluster around nucleosome dyads, linker regions, and TF footprints, recapitulating in vivo nucleosome occupancy and providing TOO information. Orientation-aware fragmentation—coverage asymmetries and phased ends around open chromatin—enables tissue deconvolution when referenced to DNase/ATAC maps [52]. End-centric features such as preferred end coordinates and 4-mer end motifs reflect nuclease usage and chromatin state, providing orthogonal signal that complements size and coverage features [53].

### 5.2. Epigenetics

#### 5.2.1. Targeted Methylation MCED

In CCGA, a targeted methylation MCED assay reached 99.5% specificity (95% CI 99.0–99.8), with overall sensitivity of 51.5% that increased with stage (≈17% I, 40% II, 77% III, 90% IV) and correct TOO/cancer-signal origin in ~89% of true positives across >50 cancer types. In the prospective PATHFINDER study with return-of-results, specificity was 99.1%, NPV 98.6%, PPV 38%, and TOO accuracy 85% for the first prediction (97% when considering first and second predictions). These data support operating at very high specificity to limit unnecessary procedures, while emphasizing stage-linked sensitivity and the practical value of TOO-guided work-up [20,54].

#### 5.2.2. Whole-Methylation Options

Enzymatic methyl-seq (EM-seq) protects 5-mC/5-hmC and selectively deaminates unmodified cytosines, preserving cfDNA integrity and improving coverage evenness at low input, while remaining compatible with bisulfite-style analysis. TAPS (TET-assisted pyridine borane sequencing) directly reads modified cytosines with higher mapping rates and more even coverage than WGBS, reporting total 5-mC + 5-hmC [55]. In plasma, cfTAPS generates methylation-based detection and TOO features together with fragment size and end/footprint information from as little as 10 ng input, enabling integrated epigenetic–fragmentomic analysis without extra assays or substantially deeper sequencing [56].

In the context of population-level cancer screening, targeted and genome-wide methylation assays represent distinct methodological trade-offs. Targeted methylation assays focus on predefined, cancer-informative regions, enabling high analytical sensitivity at substantially reduced sequencing depth and cost. This design improves scalability, robustness and cost-effectiveness, making targeted approaches more suitable for large asymptomatic populations.

In contrast, genome-wide methylation profiling captures a broader epigenetic landscape and may provide richer biological information, including tissue-of-origin inference and discovery of novel cancer-associated patterns. However, this increased information content comes at the expense of higher sequencing depth, greater computational complexity and increased cost, which currently limit its feasibility for widespread population screening. Consequently, whole-genome methylation approaches are more commonly applied in discovery studies or high-risk cohorts, whereas targeted methylation assays are preferentially adopted in clinically deployable screening workflows [44]. These trade-offs have motivated hybrid strategies that integrate targeted methylation panels with machine-learning models to balance sensitivity, specificity and scalability in multi-cancer early detection.

#### 5.2.3. cfMeDIP-Seq

cfMeDIP-seq enriches methylated cfDNA of 5-mC with antibody-based pull-down, trading base-pair resolution for high signal-to-noise at 1–10 ng input. Its bisulfite-free, short workflow is compatible with screening, and spike-in standards plus dedicated pipelines improve quantitative comparability. Clinically, cfMeDIP-seq has enabled sensitive detection and classification in foundational studies, showed high performance in RCC (plasma AUROC ~0.99; urine ~0.86 with enrichment of stage I–II), and outperformed mutation assays in intracranial tumors with low ctDNA shedding, with growing data in hereditary-risk surveillance [57].

#### 5.2.4. 5-hmC Assays

5-hydroxymethylcytosine (5-hmC) marks open, transcriptionally active chromatin, and cfDNA 5-hmC profiles carry tumor- and tissue-specific signals at low TFx. Early multi-cancer studies reported cancer-type signatures, particularly in colorectal and gastric cancer, with stage tracking. 5-hmC-Seal enables genome-wide 5-hmC profiling from 1 to 10 ng cfDNA without bisulfite-induced degradation, while retaining fragment-level information [58]. In PDAC, 5-hmC models achieved AUCs of 0.92–0.94 and captured pancreas-specific regulatory programs. In a large CRC case–control cohort (METHOD-2; *n* = 2576), a 96-gene 5-hmC model achieved an AUC of 90.7% for stage I–III CRC and detected advanced adenomas (AUC 78.6%). Fragmentomic features extracted from the same 5-hmC data further improve performance when integrated, and multimodal frameworks such as SPOT-MAS boost early-stage sensitivity and facilitate TOO inference. Overall, 5-hmC assays provide complementary signal at low input and enable built-in fragmentomics without additional WGS [59].

In addition to 5-methylcytosine (5-mC), 5-hydroxymethylcytosine (5-hmC) represents an important intermediate of active DNA demethylation and has attracted interest as a potential epigenetic signal in liquid biopsy. Compared with 5-mC, 5-hmC exhibits greater tissue specificity and more dynamic regulation, reflecting transcriptionally active genomic regions.

However, 5-hmC is present at substantially lower abundance in circulating cell-free DNA, posing significant technical challenges for sensitive and reproducible detection. Current 5-hmC profiling methods often require additional enrichment steps and lack standardized clinical workflows, limiting scalability and cross-study comparability. Consequently, despite its biological appeal, 5-hmC-based assays remain largely exploratory and have not yet demonstrated advantages over established 5-mC-based approaches for population-level cancer screening [60]. At present, 5-hmC is best regarded as a complementary epigenetic marker that may enhance biological interpretation rather than a replacement for 5-mC in clinically deployable screening assays.

### 5.3. ULP/LP-WGS for CNA and Tumor Fraction

Ultra-low-pass/low-pass WGS (ULP/LP-WGS) with ichorCNA quantifies arm-level and focal somatic CNAs and infers TFx from depth imbalances against a panel of normals using HMMcopy [61]. At ~0.1× coverage, TFx estimates are accurate when TFx > 3% (reported sensitivity 0.95, specificity 0.91), providing a pragmatic threshold for assay design; orthogonal validation has confirmed precise TFx relative to WES and prespecified 4% target. Although the earliest disease may fall below these TFx thresholds, LP-WGS is cost-efficient, scalable, and biologically specific [62].

Rapid surrogates such as RealSeqS and mFAST-SeqS approximate aneuploidy at minimal input and cost: RealSeqS amplifies ~350,000 repeat elements, whereas mFAST-SeqS yields genome-wide aneuploidy scores that correlate with ctDNA levels and outcomes across tumor types. LP-WGS at shallow coverage can still support serial monitoring of patients and analysis of subtype-specific genomic features. This requires that basic quality thresholds for coverage and variability are met and that a well-matched panel of normal samples is available [63].

## 6. Detection Platforms for ncRNA

Given that analytical workflows for ncRNAs largely parallel those for cfDNA, this section highlights platform choices that are specific to the major ncRNA classes and how they compare with cfDNA.

### 6.1. miRNA

For targeted miRNA quantification, stem–loop RT–qPCR (TaqMan) provides single-nucleotide specificity through structured RT primers and hydrolysis probes. Poly(A)-tailing RT–qPCR allows more flexible multiplexing but increases the risk of cross-reactivity without careful assay design. ddPCR offers absolute quantification and is well suited to low-abundance plasma targets.

For discovery, small RNA-seq is the standard approach; ligation bias from adapters can be reduced using randomized adapters, splint ligation or UMIs [64]. Two hybridization-based platforms avoid ligation altogether: NanoString nCounter (direct digital counting of barcoded probes) and HTG EdgeSeq (extraction-free nuclease-protection chemistry with NGS readout). Both are widely used for plasma and FFPE samples in which RNA is scarce or degraded and where reproducibility and turnaround time take precedence over breadth [65,66].

### 6.2. lncRNA

Because many lncRNAs are low copy and not all are polyadenylated, library strategy is more critical than that for cfDNA. Poly(A)+ selection yields high exonic coverage and accurate quantification for polyadenylated lncRNAs, whereas rRNA depletion captures a broader noncoding repertoire and tolerates degraded input, at the expense of deeper sequencing.

To improve sensitivity for predefined panels, hybrid-capture RNA-seq targeting curated lncRNome sets enriches low-abundance, difficult-to-detect species in human samples [67]. Long-read RNA sequencing (for example, PacBio Iso-Seq or Oxford Nanopore, including direct RNA) is increasingly used to resolve full-length transcript models without assembly ambiguity. For tissue validation and spatial biology, RNAscope RNA-ISH provides single-molecule visualization of lncRNAs in FFPE sections and is widely used for clinical-grade localization and validation [68].

### 6.3. circRNA

Short-read circRNA discovery relies on back-splice-junction (BSJ)–aware mappers; and using consensus across tools improves precision. RNase R enrichment can increase circular-to-linear ratios but is not perfectly specific. Moreover, optimized A-tailing and LiCl buffer conditions help improve enrichment and reduce artifacts [69,70]. For orthogonal confirmation and absolute quantification at the BSJ, divergent-primer qPCR and ddPCR, which is particularly useful for quantifying low copy numbers, are standard. When exon composition and internal splicing patterns are important, long-read methods directly read complete molecules and reveal circRNA-specific exon usage and retained introns. For spatial validation in tissue, BaseScope ISH uses short probe pairs spanning the BSJ to visualize specific circRNAs at near-single-cell resolution [71].

## 7. Detection Platforms for CTCs

**Figure 3:**
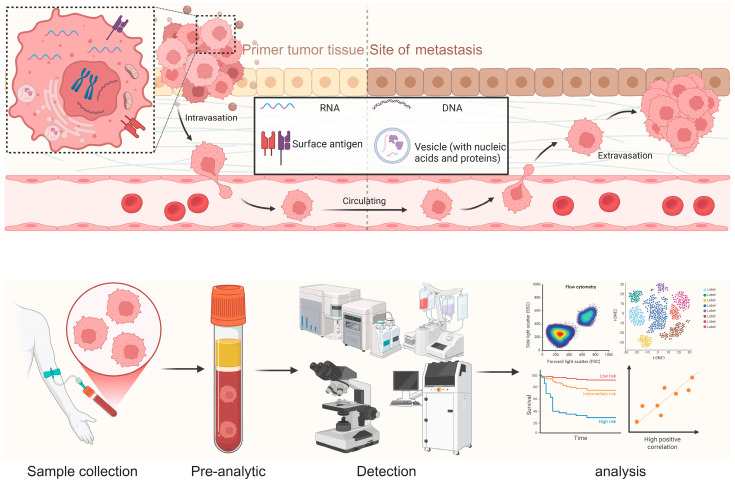
CTCs are tumor cells that detach from primary tumor tissues and enter the cardiovascular system or other biological fluids. Because the presence of CTCs is closely associated with tumor metastasis, they can provide direct and clinically relevant information about the biological characteristics of tumor tissues. Once isolated, CTC samples are subjected to downstream laboratory analyses, such as flow cytometry and single-cell RNA sequencing, enabling prognostic evaluation and comprehensive molecular characterization of tumor cells.

As a potential liquid-biopsy biomarker that carries rich information about the originating tumor tissue, CTCs have prompted the development of multiple workflows, kits and platforms (Figure 3). Below, we briefly introduce CellSearch, Parsortix, CTC-iChip, ClearCell, RareCyte and Epic, and conclude with a summary of intended-use scenarios, bias profiles and validation frameworks (Table 4).

### 7.1. CellSearch

CellSearch is the only FDA-cleared CTC enumeration system, using EpCAM-based immunomagnetic capture (ferrofluid anti-EpCAM) followed by cytokeratin (CK8/18/19), DAPI and CD45 staining; EpCAM^+^/CK^+^/DAPI^+^/CD45^−^ events are counted per 7.5 mL of whole blood. It is approved for prognostic monitoring in metastatic breast, colorectal and prostate cancer, where higher CTC counts (≥5 CTCs/7.5 mL for breast and prostate; ≥3 for CRC) predict inferior PFS and OS across pivotal trials [72]. A locked workflow ensures standardized gating and assay reproducibility and has been extensively validated in FDA 510(k) submissions, making CellSearch the reference comparator in multicenter studies. However, reliance on EpCAM/CK markers under-detects EpCAM-low, EMT or hybrid CTCs and largely misses non-epithelial phenotypes [73,74]. Routine use of CellSave preservative tubes stabilizes fixed cells for up to 96 h to aid logistics, but fixation precludes viability; for RNA-based or viable-cell analyses, EDTA blood processed within 24 h is recommended [75].

### 7.2. Parsortix

Parsortix is a label-free microfluidic system that isolates CTCs by size and deformability using a stepped cassette, retaining larger or less-deformable tumor cells and clusters while most blood cells pass through, with captured cells recovered by reverse flow for downstream analysis. In May 2022, the FDA granted De Novo Class II authorization for the Parsortix PC1 system to enrich CTCs from K_2_EDTA blood in metastatic breast cancer, limited to enrichment only; identification and diagnostic interpretation require separate validation. Analytical studies report linear recovery (~65–70%), detection of 3–5 cells per 7.5 mL, negligible carryover and compatibility with morphology, protein and nucleic-acid assays [76,77,78]. Clinically, multicenter cohorts have shown viable harvests that preserve EMT and heterogeneous phenotypes and support diverse downstream workflows (immunofluorescence, FISH/ISH, WGA–NGS, RNA panels, single-cell assays) [79,80]. Parsortix mitigates EpCAM bias but can under-recover very small or highly deformable CTCs, and performance remains indication-specific, requiring context-based validation beyond the authorized metastatic breast cancer use [76,81].

### 7.3. CTC-iChip

The CTC-iChip is an antigen-agnostic microfluidic platform that enriches CTCs through sequential physical principles: deterministic lateral displacement removes red cells and platelets, inertial focusing aligns nucleated cells, and magnetophoresis depletes leukocytes labeled with magnetic beads, yielding unlabeled CTCs including EpCAM-low and EMT phenotypes. Operating at ~10^7^ cells/s under low shear, it preserves morphology and viability for downstream assays such as immunofluorescence, FISH, WGA and NGS [82]. Although not FDA-cleared, iChip is widely used in translational studies that require minimal capture bias and live-cell recovery, and foundational work has demonstrated linear spike-in recovery and strong leukocyte depletion. Performance depends on optimized depletion antibodies and flow conditions, and residual leukocytes necessitate QC via CD45 gating, morphology filters and spike-in controls to ensure reproducibility.

### 7.4. ClearCell

ClearCell FX/FX1 is a label-free spiral microfluidic system that separates cells by size and deformability through Dean Flow Fractionation within the CTChip FR cartridge, directing larger, less-compliant CTCs and clusters to a collection outlet while removing smaller blood cells. The automated FX/FX1 instruments standardize prime–separate–collect steps and flow control, enabling reproducible, EpCAM-independent enrichment that preserves viability for culture or molecular profiling. Studies demonstrate viable CTC recovery with strong leukocyte depletion and retention of clusters and EMT-like phenotypes across multiple tumor types, with smooth transfer to immunofluorescence, FISH, targeted NGS, RNA and single-cell analyses. Advantages include high throughput, epitope-agnostic capture and compatibility with multi-omic workflows, whereas limitations include potential under-recovery of very small or highly deformable CTCs and declining purity with delayed processing, underscoring the need for prompt handling and run-to-run QC [83,84,85].

### 7.5. RareCyte

RareCyte uses a morphology-first, non-pre-enrichment workflow that minimizes capture bias. The AccuCyte system recovers the full nucleated cell fraction by density separation and spreads it as a monolayer on glass slides, which are then imaged on the CyteFinder platform using multiplex immunofluorescence (DAPI/CK/CD45 ± exploratory markers) and machine-learning-assisted candidate ranking. The CytePicker micromanipulator enables precise single-cell retrieval for downstream PCR, WES or RNA assays, allowing integration of morphology, protein and nucleic-acid data from the same specimen. Studies report ~90% spike-in recovery and single-cell detection down to 1 cell per 7.5 mL, with support for multiplex IF, FISH, WGA, targeted NGS and single-cell multi-omics across tumor types. Key advantages are minimal capture bias, high-content imaging and direct single-cell access, whereas trade-offs include a heavier image-analysis burden and reliance on robust classifiers and QC to control background and maintain specificity [86,87].

### 7.6. Epic

Epic Sciences’ platform performs direct, enrichment-free CTC analysis by plating the full nucleated cell fraction from whole blood onto proprietary slides for high-throughput immunofluorescence imaging and computational classification. CTCs are identified by quantitative morphology and phenotype, enabling detection of heterogeneous and EMT-like cells that may escape EpCAM-based capture. Multiplex panels, typically including cytokeratins, AR/AR-V7, Ki-67 and CD45, link morphology to pathway-level protein profiles. Deployed as CLIA-certified laboratory-developed tests, the platform underpins the validated AR-V7 nuclear protein assay that guides taxane versus AR-targeted therapy selection in metastatic castration-resistant prostate cancer, with consistent prognostic performance across studies [88,89]. Slide-based localization permits re-imaging, marker expansion and limited single-cell retrieval, but the platform’s primary strength lies in imaging-based biomarker assays rather than live-cell applications; limitations include higher background due to non-depletion, low viability for culture and reliance on platform-specific analytics and QC for reproducibility [90].

## 8. Detection Platforms for EVs

Although translational evidence for EVs is less mature than for cfDNA, EVs offer distinctive value by carrying multi-omic signals and enabling longitudinal monitoring. In the following sections, we review major isolation strategies—size-exclusion chromatography (SEC), ultracentrifugation (UC) and density gradients, tangential-flow filtration (TFF), immunoaffinity capture and PEG precipitation—and readout platforms, including nanoparticle tracking analysis (NTA), microfluidic or solid-state resistive-pulse sensing (MRPS/TRPS), small-particle flow cytometry (nano-flow) and single-particle interferometric reflectance imaging (SP-IRIS) (Table 5). We then summarize bias-control measures and reporting requirements in line with MISEV and EV-TRACK guidelines.

### 8.1. Size-Exclusion Chromatography

Size-exclusion chromatography separates EVs from soluble proteins and lipoproteins based on size, with lipid-bilayer vesicles eluting earlier than smaller molecules. In plasma, well-tuned SEC yields cleaner proteomic and RNA profiles than ultracentrifugation or PEG precipitation, with acceptable recovery of small EVs [91,92]. Although not IVD-cleared, SEC is endorsed by ISEV as a high-specificity plasma EV method and underpins many clinical proteomic and transcriptomic studies. Transparent reporting of column type, pore size, flow conditions and reconcentration procedures is important for cross-study comparability.

### 8.2. Ultracentrifugation and Density Gradients

Ultracentrifugation and density-gradient UC isolate EVs by sedimentation and buoyant density. In plasma, EVs typically band at 1.08–1.19 g/mL, overlapping with lipoproteins and protein complexes, so gradient composition and fractionation must be carefully optimized [93,94]. Properly tuned iodixanol or sucrose gradients can yield high-purity fractions suitable for EM, proteomics and RNA analysis, but protocols are labor-intensive and sensitive to rotor geometry, g-force and braking conditions [95]. Because co-isolation of non-vesicular components is common, many workflows now combine UC with SEC or bind–elute cleanup to improve biochemical specificity at the cost of some yield.

### 8.3. Tangential-Flow Filtration and Ultrafiltration

Tangential-flow filtration concentrates and buffer-exchanges EVs by passing samples parallel to a semipermeable membrane, retaining vesicles while allowing small solutes to permeate. Crossflow geometry reduces clogging and enables liter-scale processing upstream of SEC or UC. For plasma, regenerated cellulose or PES membranes (typically 100–500 kDa MWCO) retain small EVs and deplete albumin and soluble proteins, although very small vesicles or nanocomplexes may partially permeate at high flux [96,97,98]. TFF is widely used as a scalable front end for EV workflows because it combines high throughput with relatively gentle handling; reporting membrane type, MWCO and key operating parameters facilitates reproducibility [99].

### 8.4. Immunoaffinity Capture

Immunoaffinity capture isolates defined EV subsets by binding surface markers such as CD9, CD63, CD81 or tumor/lineage antigens on beads, chips or resins. This yields high specificity and clean phenotyping and is well suited to single-particle or multiplex readouts such as SP-IRIS, bead-based ELISA and ExoView-type platforms [100]. However, coverage is restricted to marker-high vesicles and can under-sample marker-low or epitope-masked EVs; performance depends on antibody quality, epitope density and nonspecific adsorption. Mild elution conditions are preferred when vesicles will be used for proteomics or RNA-seq. MISEV and EV-TRACK emphasize disclosure of antibody targets and capture formats, and immunocapture is best viewed as a complement to, rather than a replacement for, non-affinity bulk isolation [101,102].

### 8.5. Polymer Precipitation (PEG-Based) and Commercial Kits

PEG-based precipitation reduces solubility of colloidal particles so that EVs and associated macromolecular complexes co-aggregate and can be pelleted or filtered after brief incubation [103]. It is attractive for its speed, high apparent yield and minimal equipment requirements compared with UC. Head-to-head studies show higher particle counts and faster turnaround than UC and reasonable small-EV recovery, but PEG also co-precipitates lipoproteins and protein aggregates, which can inflate particle counts and confound omics readouts unless followed by additional cleanup. Direct comparisons indicate that SEC-polished preparations contain less non-vesicular miRNA and more EV-associated RNA than PEG pellets, supporting the use of PEG as a front-end concentrator rather than a stand-alone purification step for discovery proteomics or transcriptomics [104].

### 8.6. Nanoparticle Tracking Analysis and NanoSight/ZetaView

Nanoparticle tracking analysis estimates EV size and concentration by tracking Brownian motion of light-scattering particles and converting diffusion coefficients into hydrodynamic diameters and counts; fluorescent NTA extends this to marker-positive subpopulations [95,105]. Because EVs have a lower refractive index than polystyrene beads, naïve bead calibration causes systematic under-counting and size bias, and over-concentrated samples can produce swarm artifacts. MISEV2023 stresses standardized acquisition, refractive-index-aware calibration and transparent reporting of settings and software to enable cross-site comparisons. NTA is widely used as a core EV characterization method, but refractive-index bias and operator dependence mean that pairing with an electrical or orthogonal counter is often advisable for absolute quantitation [106].

### 8.7. Microfluidic/Solid-State Resistive-Pulse Sensing

Resistive-pulse sensing (RPS), implemented as tunable pores or fixed microfluidic apertures, detects individual particles by transient ionic-current drops as they traverse a nanoconstriction, yielding refractive-index-independent diameters and absolute concentrations when pore geometry and driving conditions are defined. In EV analysis, pore size and pressure are tuned to reduce coincidence and clogging, with reference beads used for calibration. Studies report high precision and accurate size spectra across standards and EVs, resolving mixed populations better than optical methods and avoiding the low-RI undercounting seen in NTA [107,108]. Strengths include absolute single-particle counting, low sample volume and RI independence; limitations are lower throughput and pore sensitivity to fouling [109].

### 8.8. Nano-Flow Cytometry and Small-Particle Flow

Small-particle flow cytometry adapts scatter triggering and ultrasensitive fluorescence detection to the single-EV range, enabling multi-marker immunophenotyping with MESF/ERF-traceable calibration [110]. Current instruments detect particles roughly from 40 to 1000 nm and report both scatter (as a size proxy) and fluorescence, but accuracy depends critically on calibration and instrument alignment [111]. Nano-flow delivers high-throughput, multiplexed phenotyping and is now a leading single-particle tool; calibration complexity and sensitivity to swarm artifacts mean that many studies cross-check nano-flow data against electrical or imaging methods for absolute counting and verification [112].

### 8.9. Single-Particle Interferometric Reflectance Imaging

Single-particle interferometric reflectance imaging sensing captures EVs on antibody-coated surfaces and measures their label-free interferometric scattering, with optional fluorescence channels to assess marker co-expression on the same vesicle [111]. It enables per-spot particle counts and single-vesicle multiplexing directly from plasma, and CD9/CD63/CD81-positive EV readouts correlate well with nano-flow when absolute concentrations are cross-checked by electrical or optical counters. Capture-epitope dependence and chip-to-chip variability require careful control, and current MISEV/EV-TRACK guidance emphasizes detailed reporting of chip type, surface chemistry and acquisition settings. SP-IRIS is currently research-use only but is increasingly applied in translational studies for single-EV tetraspanin profiling and heterogeneity mapping [113].

### 8.10. Electron Microscopy and Ultrasensitive Bulk Immunoassays

Electron microscopy (negative-stain or cryo-EM) provides visual confirmation of lipid-bilayer vesicles and helps identify impurities such as protein aggregates and lipoproteins. MISEV emphasizes that EM is not quantitative and should be interpreted in the context of representative, low-magnification fields and clearly described imaging conditions [111,114]. Cryo-EM further improves discrimination of bona fide EVs from non-vesicular particles and supports morphology-based purity assessment [115]. Ultrasensitive digital immunoassays such as Simoa extend bulk detection of EV markers or tumor antigens to very low concentrations, enabling longitudinal tracking when antibodies are well characterized and matrix effects are controlled [116,117]. Because bulk protein signals can originate from non-vesicular carriers, current guidance encourages combining morphology (EM), biophysical particle counts and bulk protein measurements, reported with sufficient metadata to support replication [111,118]. In practice, EM anchors structural identity, whereas assays such as Simoa provide clinical-scale sensitivity for predefined antigens; both are most informative when interpreted alongside single-particle metrics.

## 9. Key Limitations and Barriers to Widespread Clinical Implementation

Despite substantial advances in liquid biopsy technologies, multiple biological, technical, analytical and clinical barriers continue to limit their widespread implementation in population-level cancer screening and early detection. Although these limitations vary across analytes and assay platforms, they collectively constrain achievable sensitivity, specificity, scalability and clinical interpretability, particularly in asymptomatic populations (Table 6). Here we synthesize current limitations from the analyte-specific discussions above.

### 9.1. Low Abundance and Specificity of Tumor-Drived Signals in Stage I-II Cancers

From a biological perspective, a central challenge is the extremely low abundance of tumor-derived signals in early-stage disease. In stage I–II cancers, the fraction of tumor-derived DNA in circulation often falls below the detection threshold of current technologies, while circulating tumor cells are frequently absent or present at exceedingly low frequencies [10,24]. Similarly, tumor-derived extracellular vesicles and non-coding RNAs represent only a small subset of total circulating material, imposing a biological ceiling on sensitivity that cannot be fully overcome through technical optimization alone. Moreover, many circulating signals lack tumor specificity. cfDNA abundance, fragmentation patterns and epigenetic features can be influenced by aging, chronic inflammation, tissue injury and benign pathological conditions. Age-associated clonal hematopoiesis introduces somatic mutations unrelated to malignancy, substantially increasing the risk of false-positive findings in screening settings [119].

### 9.2. Tumor Heterogeneity and Variation in Shedding Dynamics

Tumor heterogeneity and variable shedding dynamics further complicate interpretation. Different tumor types, stages and molecular subtypes exhibit distinct release behaviors into the circulation, and some localized tumors may release little or no detectable material at early stages [120]. Consequently, reliance on a single biomarker or analyte is unlikely to yield uniform sensitivity across cancer types, limiting the feasibility of universal screening strategies.

### 9.3. Difficulty in Enrichment and Detection of Low Input Analytes

Technical and experimental limitations constitute additional major barriers. Detection of rare analytes such as circulating tumor cells and extracellular vesicles depends on enrichment procedures that introduce systematic bias and variability. EpCAM-based CTC capture approaches may fail to recover cells undergoing epithelial–mesenchymal transition, while size-based or immunoaffinity-based EV isolation methods often yield heterogeneous vesicle populations with variable purity, compromising assay reproducibility and cross-study comparability [114,121]. Furthermore, low-abundance signals—including 5-hydroxymethylcytosine and EV-associated RNAs—are highly sensitive to pre-analytical variables such as sample handling, extraction efficiency and batch effects, resulting in limited robustness across laboratories.

### 9.4. Lack of Standardized and Clinical Scalable Workflows

A lack of standardized, clinical-grade workflows further restricts scalability. Substantial heterogeneity exists across studies with respect to sample collection protocols, cfDNA extraction methods, library preparation strategies and bioinformatic pipelines [6]. For genome-wide methylation profiling and multi-omics assays, the requirement for high sequencing depth and complex data processing substantially increases cost and limits throughput, posing challenges for deployment in large, population-based screening programs.

### 9.5. Analytical Challenges Associated with High-Dimensional Data

Analytical and interpretative challenges also remain unresolved. High-dimensional data generated by genome-wide methylation, fragmentomic and multi-omics assays necessitate sophisticated computational models and large training cohorts, raising concerns regarding model generalizability and reproducibility [122]. In many studies, statistical significance or high area-under-the-curve values are reported without corresponding effect sizes or clear links to clinical decision-making. In screening contexts, the detection of molecular abnormalities does not necessarily translate into actionable clinical outcomes, complicating risk stratification and downstream management.

### 9.6. Limitations in Clinical Verification and Translational Implementation

Finally, critical gaps persist in clinical validation and translational evidence. Most available data are derived from retrospective or case–control studies, whereas large-scale prospective screening cohorts in asymptomatic populations remain scarce [11]. Direct head-to-head comparisons across different analytes and platforms within the same cohorts are uncommon, limiting objective assessment of relative performance. Moreover, standardized clinical pathways for managing positive liquid biopsy findings—including lesion localization, confirmatory testing and avoidance of overdiagnosis—have yet to be fully established.

Collectively, these biological, technical and clinical constraints explain why no single liquid biopsy modality has yet achieved broad applicability for population-level cancer screening. Addressing these challenges will likely require multimodal integration, rigorous standardization and carefully designed prospective validation studies before widespread clinical deployment can be realized.

## 10. Multimodal AI: Deep-Learning Fusion with Radiomics

Deep learning strengthens liquid biopsy by amplifying weak, low–tumor-fraction signals from cfDNA fragmentomics and methylation and fusing them with radiomic descriptors that carry spatial and organ context. Across multicenter, externally validated cohorts, such late-fusion designs achieve screening-grade specificity while improving PPV and reducing unnecessary procedures. A pathway-aware workflow then follows naturally, with pre-specified referral thresholds and follow-up intervals.

Multimodal AI that fuses liquid biopsy with imaging has advanced rapidly. In ASCEND-LUNG, a prospective two-stage pipeline links a blood-based multi-omics screen (cfDNA methylation plus proteins) to an AI nodule classifier that integrates CT features with methylation. In independent validation, the screen reached an AUC of 0.963 with 99% sensitivity at 56% specificity, and the nodule model achieved 81.1% sensitivity and 76.0% specificity, illustrating an end-to-end, clinically minded workflow from pre-LDCT triage to post-LDCT management [123]. Complementary work shows that adding simple CT signs and age to cfDNA methylation panels improves nodule risk stratification and supports a practical two-threshold deployment strategy that curbs overtreatment while preserving accuracy [124]. For small (5–10 mm) nodules, a deep-learning model that jointly learns cfDNA methylation, plasma proteins and CT radiomics outperforms any single modality (test AUC 0.925 overall; AUC 0.951 in 5–10 mm nodules) [125]. Beyond methylation, integration of radiomics with cfDNA fragmentomic and epigenomic signals is being accelerated by a new multicenter “radio-epigenomic” dataset pairing chest CT with plasma cfDNA end-motif profiles from more than 2000 patients, providing a robust benchmark for model development and external testing [126]. In the therapy setting, combining mid-treatment ctDNA kinetics with baseline radiomics improves progression-free-survival prediction in CRT-treated NSCLC, pointing to response-adaptive care pathways [127]. Complementing these lung-focused efforts, in 2025, Yin et al. developed a cfDNA fragmentomics-based early-detection model for PDAC using shallow-WGS features (fragment size, CNV, mutational and methylation signatures) and reported high accuracy across training, internal validation (AUC 0.987) and two external cohorts (91% sensitivity at 94.5% specificity), providing a strong molecular backbone that could be fused with CT/MRCP/EUS radiomics in future multimodal PDAC frameworks [128]. Finally, multimodal networks that combine CT with CTC readouts are beginning to show value for mediastinal tumor classification, while deep-learning-based CTC detectors improve input fidelity for such pipelines [129,130].

Beyond discrimination metrics such as AUROC, multimodal studies should also report calibration and decision-curve analyses, PPV/NPV at fixed specificity and tissue-of-origin accuracy. Methodologically, UMI/duplex error suppression, blinded data splits and site-shift external validation help prevent information leakage and improve generalizability. With these guardrails in place, deep learning is best viewed not as a replacement for radiology or genomics, but as the glue that operationalizes both for screening, diagnostic work-up and surveillance.

## 11. Conclusions

At present, cfDNA remains the most mature, extensively studied and broadly applicable analyte for liquid biopsy, supported by the strongest body of analytical and clinical evidence. Most contemporary cfDNA-based studies focus on integrative multi-omic frameworks—such as methylomics and fragmentomics—to construct predictive models with improved sensitivity and accuracy. While these approaches continue to advance, the incremental performance gains achieved through cfDNA alone appear to be diminishing under current analytical paradigms, prompting increasing interest in alternative analytes, including circulating tumor cells and extracellular vesicles. In contrast to cfDNA, CTC- and EV-based approaches face distinct challenges, with technical limitations in isolation, standardization and scalability remaining the primary barriers. Nevertheless, when successfully detected, these analytes can provide high-quality biological evidence with strong mechanistic relevance.

Moving beyond mutation-centric testing, the field is increasingly shifting toward multimodal decision frameworks centered on cfDNA fragmentomic and epigenetic signals, complemented by analytes such as circulating tumor cells and extracellular vesicles. Accumulating evidence indicates that these non-mutational features preserve tissue and chromatin context even at low tumor fractions, thereby offering more stable and informative signals than mutation-based assays alone. When integrated with advanced computational approaches and, in selected contexts, imaging-derived features, such multimodal strategies have the potential to support blood-first, high-specificity risk stratification, improving positive predictive value and reducing unnecessary diagnostic procedures while maintaining clinically acceptable sensitivity. Importantly, translating analytical discrimination into clinical utility requires methodological rigor and deployment-aware evaluation, including standardized pre-analytical workflows, robust error-control strategies and clinically coherent integration pathways linking blood-based risk signals to downstream diagnostic assessment. Looking ahead, progress in this area is likely to depend less on isolated algorithmic performance and more on system-level validation. Prospective studies in real-world and health-system settings will be essential to assess the net clinical impact of blood-first triage strategies, including effects on diagnostic yield, time to diagnosis and patient burden. As these elements mature, liquid biopsy approaches may evolve from signal discovery tools into clinically integrated, multimodal systems supporting screening, diagnostic work-up and longitudinal disease surveillance.

## Figures and Tables

**Table 1:** Detection platforms used for analytes and their advantages.

Analyte	Primary Source/Biogenesis	Representative Detection Platforms	Key Strengths	Typical Clinical Applications
cfDNA/ctDNA	Apoptosis, necrosis, NETosis, and active secretion from tumor and non-tumor tissues; circulates in plasma as short fragments.	ddPCR/BEAMing; targeted NGS (CAPP-Seq, TAm-Seq, TEC-Seq); WES/WGS; methylation assays (WGBS/ERRBS, cfMeDIP-seq); 5hmC-seq; DELFI-like fragmentomics.	Most mature multi-omic analyte; highly specific signals; scalable; compatible with MRD and longitudinal monitoring.	MCED screening (research/early pilots), MRD detection, therapy monitoring, tissue-naïve genotyping when tissue is unavailable.
CTCs	Intact malignant cells shed from primary tumors and metastases into blood.	Immuno-enrichment (EpCAM: CellSearch); label-free size/deformability (Parsortix, ClearCell, CTC-iChip); imaging cytometry; scRNA/WGA-WGS.	Cellular context enables pathway, phenotype, and functional testing; resolves spatial heterogeneity.	Prognosis, therapy selection, metastasis biology, minimal use in general screening.
Extracellular vesicles	Exosomes (endosomal), microvesicles (plasma-membrane budding), apoptotic bodies; released by tumor and stromal cells.	Isolation: differential UC, SEC, TFF, PEG precipitation, immunocapture; Readouts: NTA, MRPS, SP-IRIS, nano-flow; Omics: EV-RNA-seq, proteomics; single-EV assays.	Cargo stability; TOO information; abundant particles; suited for multi-omic panels	Adjunctive early detection signatures (EV-miRNA/protein), disease monitoring, treatment response tracking.
Tumor-educated platelet	Platelets reprogrammed by tumor-derived signals (direct contact, EV uptake, cytokines), showing altered RNA splicing and content.	Standardized platelet isolation; RNA-seq/qPCR panels; machine-learning classifiers for cancer vs. healthy and tissue-of-origin.	Very high abundance; robust RNA yield; informative TOO signals; low input volume.	Research-stage early detection and triage; disease monitoring.
Circulating linear ncRNAs (miRNA/lncRNA)	Released free, EV-encapsulated, or protein-boun from tumor and microenvironmental cells.	RT-qPCR panels; small-RNA-seq; microarrays, often with spike-ins and hemolysis controls.	Stable in biofluids; relatively low cost; panels feasible for large cohorts.	Adjunctive screening and diagnosis; risk stratification; longitudinal monitoring.
Circulating circRNA	Back-spliced circular RNAs from tumor and stromal cells; found free and within EVs.	RNase-R enrichment; circRNA-aware RNA-seq mapping; junction-specific RT-qPCR; orthogonal validation (Sanger/RNase-R).	Exceptional stability; potential tissue-specificity; emerging as complementary RNA analyte.	Candidate biomarkers (GI, lung, HCC) in discovery and validation stages.

**Table 2:** Principles, advantages and disadvantages of different detection platforms for cfDNA.

Platform	Principle	Advantages	Disadvantages
WGS (shallow, deep)	cfDNA libraries without capture/targeting; shallow WGS supports genome-wide signatures; deep WGS for high-resolution follow-up.	Fragmentation/CNA signatures have emerged as useful biomarkers for detection.	Trade-offs implied by depth.
WGBS	Base-resolved genome-wide 5-mC profiling of cfDNA.	Base-resolution methylation information across the genome.	Bisulfite conversion may introduce system errors or information loss.
Digital PCR (ddPCR, BEAMing)	Molecular partitioning into 104–105 droplets/beads with probe-based binary readout for absolute molecule counts.	Absolute quantification of targeted variants.	Not suited for broad genomic profiling; early-cycle PCR errors can propagate in comparable assays.
TAm-Seq	Adapter/UMI ligation to native cfDNA fragments enabling multiplex amplicon sequencing.	Multiplexed targeted sequencing with UMI support.	CHIP confounding requires matched WBC or stringent filters.
CAPP-seq	Hybrid-capture with UMI-aware background suppression for SNVs/indels/CNAs.	Capture-based breadth with error-suppressed detection across variant classes.	CHIP confounding requires matched WBC or stringent filters.
WES	Surveys coding regions where most pathogenic mutations occur.	Covers exome for broad variant discovery in genes of interest.	Resource-intensive even with duplex/UMI improvements.
DELFI (fragmentomics from shallow WGS)	Position-dependent size/coverage imbalances reflect nucleosome architecture; shallow WGS input.	Demonstrated balanced sensitivity/specificity with favorable PPV/NPV in high-risk screening populations.	Reliance on population-scale training and pre-analytic control
ULP/LP-WGS with ichorCNA	Quantifies arm-level/focal SCNAs and estimates tumor fraction (TFx) from shallow WGS.	Highly specific (aneuploidy is rare in non-malignant states).	Sensitivity limitations at very low TFx addressed by pairing.
RealSeqS/mFAST-SeqS	Rapid surrogates approximating aneuploidy from shallow-coverage sequencing.	Operationally rapid aneuploidy surrogates.	Pairing with dense methylation/fragmentomics is advised in the draft.

**Table 4:** Workflows or kits currently used for detection of CTCs.

Platform	Enrichment Principle	Advantages	Disadvantages
CellSearch	EpCAM immunomagnetic positive selection; IF gating CK8/18/19^+^/DAPI^+^/CD45^−^.	Harmonized workflow; cross-study comparability.	EpCAM/CK dependence under-captures EMT/epithelial-low CTCs; fixation limits viability.
Parsortix	Label-free microfluidic size/deformability capture in stepped cassette.	Epitope-agnostic; preserves EMT/heterogeneity; gentle harvest.	May under-recover very small/highly compliant CTCs; purity degrades with delays; validate per indication.
CTC-iChip	Antigen-agnostic.	Minimizes capture bias; yields viable, heterogeneous CTCs.	Requires robust antibody cocktail and tuning; residual leukocytes require gating and QC.
ClearCell	Label-free spiral microfluidics (Dean Flow Fractionation) in CTChip FR.	Epitope-agnostic, fast, culture-friendly.	Biophysical thresholding may miss very small/highly deformable CTCs; purity declines with processing delay.
RareCyte	No affinity capture pre-enrichment; density recovery then whole nucleated cells plated as monolayer slides.	Minimal pre-capture bias; rich imaging and molecular follow-up.	Higher image-analysis burden; relies on validated classifiers and QC.
Epic	No pre-enrichment; entire nucleated fraction plated; high-throughput IF and computational classification.	No capture bias; scalable imaging analytics; strong clinical precedent via AR-V7.	Larger background search space (no depletion); platform-specific pipelines and QC required.

**Table 5:** Workflows or kits currently used for detection of EVs.

Platform	Measures and Outputs	Advantages	Disadvantages
Nanoparticle tracking analysis (NTA; NanoSight/ZetaView)	Tracks Brownian motion for size distribution and number concentration; optional fluorescence channels for marker-positive fractions.	Ubiquitous; accessible; historical comparability; fluorescence add-ons.	Optical RI bias undercounts lipid vesicles; swarm inflates counts; operator-sensitive.
Resistive-pulse sensing (RPS: MRPS/TRPS; Spectradyne nCS1/ARC; Izon Exoid)	Electrical pulses through nanopores for RI-independent diameter and absolute concentration.	Absolute single-particle counts; resolves mixed modes; small sample volumes; avoids low-RI undercounting.	Throughput lower than optical; pore/cartridge health and coincidence require QC.
Nano-flow cytometry/small-particle flow (NanoFCM, Apogee)	Single-EV scatter and multi-color fluorescence with MESF/ERF-traceable calibration; per-vesicle marker distributions.	High-throughput single-EV phenotyping; multi-parameter immunoprofiling.	Requires rigorous calibration; susceptible to swarm/coincidence in protein-rich matrices.
SP-IRIS/ExoView (single-particle interferometric reflectance imaging)	Antibody microarrays capture EVs; label-free interferometric counts co-registered with multiplex IF on the same vesicle.	Single-particle multiplexing; strong matrix tolerance; co-localization at single-EV level.	Capture-epitope dependence; chip/lot variability; research-use.
Electron microscopy (TEM/cryo-EM)	Morphology of lipid-bilayer vesicles; documents impurities.	Structural confirmation; cryo-EM preserves ultrastructure and aids bona fide EV discrimination.	Labor/interpretation burden; selection bias if fields not representative.
Ultrasensitive bulk immunoassays (Simoa)	Digital immunoassays for EV markers/tumor antigens in bulk or SEC fractions; longitudinal tracking.	Clinical-scale sensitivity; separate EV-associated vs. free proteins with proper design/calibration.	Antibody specificity/matrix effects; bulk signals can include non-vesicular carriers and need orthogonal checks.

**Table 6:** Limitations in currently used analytes and detection methods.

Limitation Category	Affected Analytes	Core Issues	Underlying Cause	Impacts
Low abundance of tumor-derived signals in early-stage disease	cfDNA, CTCs, EVs, ncRNAs	Tumor-derived signals often below detection threshold in stage I–II cancers	Limited tumor burden and weak shedding into circulation	Low sensitivity and high false-negative rates in early detection
Limited tumor specificity of circulating signals	cfDNA, epigenetic markers	Circulating signals are influenced by non-malignant conditions	Aging, chronic inflammation, tissue injury, and clonal hematopoiesis	Increased false-positive risk and reduced clinical interpretability
Tumor heterogeneity and variable shedding dynamics	All analytes	Inconsistent release of tumor material across cancer types and stages	Biological heterogeneity and anatomical constraints	Single-analyte assays fail to achieve uniform sensitivity across cancers
Difficulty in enrichment and detection of rare analytes	CTCs, EVs, low-abundance epigenetic signals	Enrichment-dependent detection introduces bias and variability	EMT-related marker loss, heterogeneous vesicle populations, low input material	Poor reproducibility and limited cross-study comparability
Sensitivity to pre-analytical variables	EVs, 5-hmC, EV-associated RNAs	Low-abundance signals are highly vulnerable to handling and batch effects	Sample collection, storage, extraction efficiency	Reduced robustness and limited inter-laboratory reproducibility
Lack of standardized clinical-grade workflows	cfDNA, multi-omic assays	Substantial heterogeneity in protocols and pipelines	Non-uniform sample processing and bioinformatic strategies	Limited scalability and difficulty in large population deployment
High cost and limited throughput of genome-wide assays	Methylomics, multi-omics	Requirement for deep sequencing and complex computation	High sequencing depth and analytical burden	Economic and logistical barriers to screening-scale application
Analytical challenges of high-dimensional data	Multi-omic and AI-based models	Model overfitting and limited generalizability	High dimensionality and insufficient training cohorts	Uncertain real-world performance and reproducibility
Limited clinical validation in asymptomatic populations	All analytes	Evidence largely derived from retrospective or case–control studies	Lack of large prospective screening cohorts	Unclear clinical utility and risk of overdiagnosis
Absence of standardized downstream clinical pathways	All analytes	Positive results lack uniform diagnostic and management workflows	Incomplete integration with imaging and confirmatory testing	Reduced actionability and clinical adoption

## Data Availability

No new data were created or analyzed in this study. Data sharing is not applicable to this article.
